# Clinical and genetic characteristics in a central-southern Chinese cohort of early-onset Alzheimer's disease

**DOI:** 10.3389/fneur.2023.1119326

**Published:** 2023-03-27

**Authors:** Zhihou Liang, Yan Wu, Chuanzhou Li, Zhijun Liu

**Affiliations:** ^1^Department of Neurology, Union Hospital, Tongji Medical College, Huazhong University of Science and Technology, Wuhan, China; ^2^Department of Medical Genetics, School of Basic Medicine, Tongji Medical College, Huazhong University of Science and Technology, Wuhan, China

**Keywords:** early-onset AD, *APP*, *PSEN1*, *PSEN2*, phenotype heterogeneity

## Abstract

**Background:**

Mutations in the presenilin-1 (*PSEN1*), presenilin-2 (*PSEN2*), and amyloid precursor protein (*APP*) genes have been commonly identified in early-onset Alzheimer's disease (EOAD). Some of the mutations in the three causative genes, especially the *PSEN1* gene, result in variable phenotypes and exhibit clinical heterogeneity among EOAD families.

**Methods:**

Using next-generation sequencing (NGS), we performed genetic screening in a Chinese cohort of 18 patients with EOAD, consisting of five familial EOAD and 13 sporadic cases.

**Results:**

We identified two likely pathogenic *PSEN1* mutations (one novel) and a novel *APP* mutation in three cases of EOAD, where two are familial and one is sporadic, respectively. In addition, we detected a few variants of uncertain significance (VUS) in several genes, including not only the two known variants in *PSEN2* (p.H169N and p.V214L) but also genes causal of other types of dementia or previously identified as risk factors for AD, suggesting the possible involvement of multiple genes in the etiopathology of AD. The patients carrying *PSEN1* mutations had an earlier mean age at the onset than those with *PSEN2* or *APP* variants. The initial symptoms varied greatly among patients in the EOAD cohort, from progressive memory impairment and epilepsy to uncommon motor symptoms such as involuntary tremors in the upper extremities.

**Conclusions:**

In conclusion, our study provides further evidence of the genetic profile of patients with EOAD from China and expands the mutation spectrum of both *PSEN1* and *APP*. In addition, our results highlight the clinical heterogeneity in patients with EOAD and mutations in *PSEN1, PSEN2*, and *APP* and suggest strong effects of genetic variants on clinical phenotypes. Future functional studies are needed to clarify the interaction between AD-causative gene mutations and phenotypic heterogeneity.

## 1. Introduction

Alzheimer's disease (AD) is a devastating neurodegenerative disease, characterized by progressive memory loss and deficits of other cognition domains that may impair daily functioning as well as define pathological changes with abnormal extracellular aggregates of amyloid-β and hyperphosphorylation of neuronal tau. AD is the most common cause of dementia, affecting ~44 million people worldwide (1). According to the age of onset, AD can be divided into two subtypes: early-onset AD (EOAD) and late-onset AD (LOAD). EOAD is typically defined as onset occurring before the age of 65 years and accounts for ~5–10% of all cases of AD, while LOAD represents the much more common type of dementia affecting individuals aged 65 years and above. Patients with EOAD tend to display an atypical clinical presentation (e.g., visual dysfunction, aphasia, executive dysfunction, apraxia, and spastic paraparesis) (2) and have a more aggressive disease progression and a shorter survival time than patients with LOAD (3). The exact pathogenic mechanisms that underlie AD remains largely unknown, and no effective disease-modifying therapeutic interventions are currently available for AD.

Both LOAD and EOAD have a high heritability, estimated to be 70–80% and 92–100%, respectively (4). Approximately 90–95% of patients with EOAD appear to be sporadic, whereas the remaining 5–10% of cases are familial and have dominant inherited mutations in amyloid protein precursor (*APP*), presenilin-1 (*PSEN1*), and presenilin-2 (*PSEN2*). Mutations in all three genes are known to cause enhanced production and deposition of amyloid beta (Aβ). Advances in next-generation sequencing (NGS) and its wide applications enable the rapid identification of a growing number of pathogenic variants or mutations associated with AD. To date, more than 450 mutations have been reported in the three causative genes that result in familial AD (FAD). Among them, *PSEN1* gene mutations are responsible for ~75% of genetic EOAD cases (https://www.alzforum.org/mutations). However, cases of high-penetrant mutations in these three genes could explain only 5–10% of EOAD cases, leaving a large group of EOAD families unexplained genetically (5). The genetics of LOAD are much less well-understood compared to EOAD, with a highly polygenic architecture of complex traits. The ε4 allele of the apolipoprotein E (*APOE*) gene has been recognized as the strongest genetic risk factor for LOAD, with a 2–3 times increase in risk in heterozygous and a 15 times increase in homozygous (5). More recently, a large genome-wide association study (GWAS) has identified 75 independent risk loci for AD and related dementias (ADD), of which 33 have been previously reported to be associated with ADD (6). Pathway-enrichment analysis supported the involvement of microglia in AD, perhaps by modulating Aβ aggregation/degradation (6).

Genotype–phenotype relationships have previously been examined for *APP* (7), *PSEN1* (8, 9), and *PSEN2* mutations (10), but at present, no clear pattern of genotype–phenotype correlation has been well-established in AD. In general, the age at onset (AAO) of AD is relatively consistent within families carrying the same FAD-linked mutations but differs markedly between individuals carrying different mutations. Patients with *PSEN1* mutations are seen to manifest an early symptom onset, with a mean AAO of 8.4 years earlier than in *APP* mutation carriers (42.9 vs. 51.3 years) and 14.2 years earlier than in *PSEN2* cases (57.1 years) (11). The mean duration of symptoms in *PSEN1*-related AD families is significantly shorter (ranging from 5.8 to 6.8 years) than in *PSEN2* (ranging from 4.4 to 10.8 years) and *APP* mutations carriers (ranging from 9.0 to 16 years), indicating that the severity of disease is associated with specific *PSEN1* mutations. Moreover, Ryan et al. (12) analyzed the genotype and phenotype correlation for autosomal dominant familial AD in the UK and Ireland populations. The researchers emphasized the phenotypic heterogeneity of familial AD and suggested that high variability in AAO and clinical presentations were determined by specific mutations as well as by causative genes (12).

Although a growing number of pathogenic mutations in *APP, PSEN1*, and *PSEN2* have been identified for EOAD families, the identification of additional EOAD-causative genes remains challenging. In this study, we conduct targeted panels and whole-exome sequencing (WES) in a cohort of patients with EOAD from the central-southern region of China, with an emphasis on the evaluation of the genetic spectrum and clinical features of patients with clinically suspected EOAD.

## 2. Materials and methods

### 2.1. Subjects

This cohort consisted of 18 patients, including 12 (66.7%) men and six women (33.3%), clinically diagnosed with EOAD with a mean onset age of 48.9 ± 6.1 years (ranging from 32 to 60 years). In total, 27.8% (5/18) of patients had a positive family history of dementia. All patients were of Han Chinese origin and were recruited from the neurology department of Wuhan Union hospital and from the central-southern region of China between March 2018 and September 2022. All affected patients were evaluated by at least two neurologists and were diagnosed according to the National Institute of Aging-Alzheimer's Association criteria (13). All patients underwent a comprehensive neurological assessment, including past medical history, family history, and neuropsychological assessment. Cerebrospinal fluid (CSF) and topographical markers (^18^F-AV-45 PET-CT) were assessed as well in 72.2% (12/18) and 22.2% (4/18) of all subjects, respectively. Written informed consent was obtained from all participants.

### 2.2. Genetic analysis

Genomic DNA was extracted from peripheral blood samples using a standard extraction method. The whole-exome sequencing (WES) and targeted panels comprising 168 genes potentially associated with AD and other types of dementia were used to perform comprehensive genomic testing. Sequencing was performed using the Illumina Hiseq2000/2500 system. Sequenced data analysis was carried out as described previously (14). Sanger sequencing was used to validate the candidate variants after variant calling and filtering. Co-segregation analysis of variants was performed in the families with available DNA samples.

## 3. Results

### 3.1. Genetic findings and pathogenicity classification of variants

**Figure 1:**
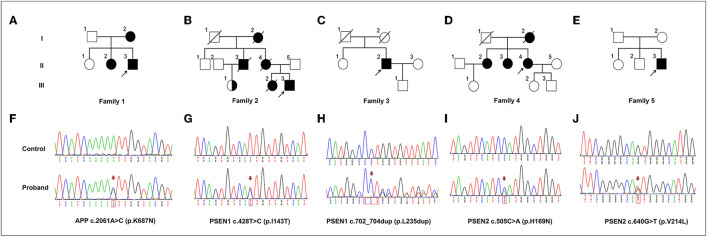
The variants in APP, PSEN1, and PSEN2 genes identified in this study. **(A–E)** Pedigrees carrying the variants in APP, PSEN1, and PSEN2. Arrows indicate the proband of each family. **(F–J)** Sequencing chromatograms of the identified variants in APP (p.K687N), PSEN1 (p.I143T and p.L235dup), and PSEN2 (p.H169N and p.V214L). Arrows indicate the variant sites.

In general, ~98.57% of the target bases were covered with at least 30 × per individual, and the mean depth of coverage for all target regions was 177.39. Only those protein function-altering variants with a low minor allele frequency in the datasets from 1,000 Genomes, ESP5400, and ExAC were retained after filtering and were assumed to be pathogenic. Finally, we identified five potentially pathogenic variants in *APP, PSEN1*, and *PSEN2*, including a novel pathogenic *APP* mutation (c.2061A > C, p.K687N), two pathogenic *PSEN1* mutations (p.I143T and p.L235dup), and two known variants of uncertain significance (VUS) in *PSEN2* (p.H169N, and p.V214L) in five unrelated EOAD families (Table 1). All of these variants were confirmed by Sanger sequencing (Figure 1). The novel mutation (p.L235dup) in *PSEN1* was not found in the public genetic variant database. Segregation analysis revealed that the two known missense variants (*APP* p.K687N and *PSEN2* p.H169N) were co-segregated through their other family members. In addition, we also detected another three VUS in several autosomal dominant genes which can lead to other types of dementia or are recognized as risk factors for AD, such as *GRN* and *SORL1* (Table 1).

**Table 1:** Rare variants in AD-related genes identified in this study.

**Mutations**				**Bioinformatics prediction**		**Population frequency**			**ACMG**	**CADD**	**References**
**Gene**	**Refseq NM**	**Nucleotide**	**Amino acid**	**Polyphen2**	**SIFT**	**ESP6500**	**GnomAD**	**ExAC**			
APP	NM_000484.4	c.2061A>C	p.K687N	Probably damaging	D	0	0	0	Likely pathogenic	11.82	This study
PSEN1	NM_000021.4	c.428T>C	p.I143T	Probably damaging	D	0	0	0	Likely pathogenic	27.5	(15)
PSEN1	NM_000021.4	c.702_704dup	p.L235dup	NA	NA	0	0	0	Likely pathogenic	NA	This study
PSEN2	NM_000447.3	c.505C>A	p.H169N	Probably damaging	D	0	0.000191	0.002311	VUS	25.2	(16)
PSEN2	NM_000447.3	c.640G>T	p.V214L	Probably damaging	D	0	0.000107	0.002543	VUS	24.4	(17)
GRN	NM_002087	c.453del	p.M152Cfs*104	NA	NA	0	0	0	VUS	NA	This study
GRN	NM_002087	c.1690C>T	p.R564C	Probably damaging	D	0	0.0001	0	VUS	0.058	This study
SORL1	NM_003105.6	c.4538C>A	p.T1513N	Possibly damaging	T	0	0.000004	0.00002	VUS	1.4	This study

PolyPhen-2, Polymorphism Phenotyping v2; SIFT, Sorting Intolerant From Tolerant; ESP6500, Exome Sequencing Project v.6500; GnomAD, Genome Aggregation Database; ExAC, Exome Aggregation Consortium; ACMG, American College of Medical Genetics; VUS, Variants of Uncertain Significance; D, Deleterious. T, Tolerated. NA, Not applicable.

### 3.2. *In silico* protein structure prediction of novel variants

**Figure 2:**
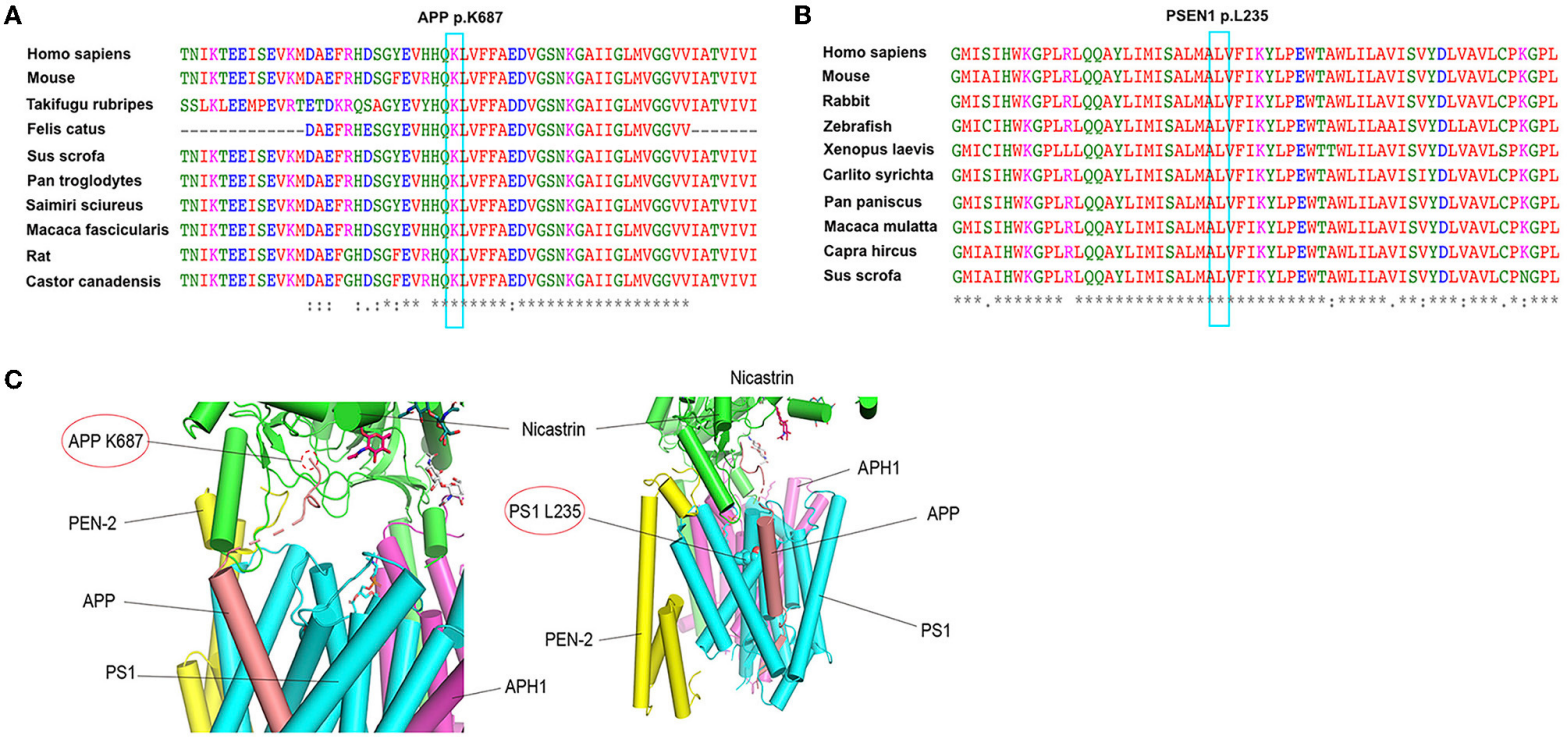
Conservation analysis of the two mutations (APP K687N and PSEN1 L235dup) and protein structure prediction. **(A)** Alignment of APP sequences from different species. Arrow indicates the mutation site. **(B)** Alignment of PSEN1 sequences from different species. Arrow indicates the mutation site. **(C)** The possible effects of two mutations, APP K687N and PSEN1 L235dup, using the complex structure of Amyloid Precursor Protein and Human gamma-secretase (PDB ID: 6IYC).

We analyzed the possible effects of mutations using the complex structure of amyloid precursor protein and human gamma-secretase (PDB ID: 6IYC) (Figure 2). The structure model suggests that the K687N mutation in *APP* likely impairs the direct interaction with nicastrin in the complex. The L235 residue in presenilin-1 is embedded inside the transmembrane domain and is close to the binding APP; as a result, the duplication of PS1-L235 residue may affect the stability of the transmembrane complex or the processing of APP *via* direct binding.

### 3.3. Clinical features of patients with likely pathogenic variants or variants of uncertain (or unknown) significance in *APP, PSEN1*, and *PSEN2*

Of the five patients with identified mutations in *APP, PSEN1*, and *PSEN2*, the mean AAO was 44.8 ± 7.9 years and 40% (2/5) of patients were women. The clinical characteristics of these patients are summarized in Table 2. The main clinical presentations of EOAD in our cohort include memory impairment, psychosocial and behavioral problems, epilepsy, and rare tremors, consistent with other previously reported EOAD cases. However, there is no difference in the amyloid/tau profile between cases of familial AD and others with sporadic AD. The clinical manifestations of patients are described in detail later.

**Table 2:** The clinical characteristics and genetic analysis of five probands carrying mutations in APP, PSEN1, and PSEN2.

**Proband no**	**Sex**	**Family history**	**Age at onset (y)**	**Age at present (y)**	**Cognitive symptoms**	**Behavioral and psychiatric symptoms**	**Additional features**	**Neuroimaging features**	**Gene**	**Variants**	**APOE**
1	M	Yes	52	53	Memory decline	-	Involuntary tremors	Enlarged ventricles and sulci, diffuse amyloid plaque deposition in the cerebral cortex	*APP*	c.2061A>C p.K687N	E3/E4
2	M	Yes	32	37	Progressive memory decline, disorientation, executive dysfunction	Apathetic, social disinhibition	Seizures	Bilateral temporal atrophy and ventricular enlargement	*PSEN1*	c.428T>C p.I143T	E3/E4
3	M	No	43	45	Memory decline, disorientation, executive dysfunction	Paranoid, apathetic, and irritable	-	Temporal atrophy and ventricular enlargement, prominent amyloid deposition in the cerebral cortex	*PSEN1*	c.702_704dup p.L235dup	E3/E3
4	F	Yes	50	54	Progressive memory decline, language disability	Apathic	Action tremors	Atrophy of cerebral cortex, predominantly in temporoparietal lobe	*PSEN2*	c.505C>A p.H169N	E3/E3
5	F	No	47	49	Memory disturbance	Apathetic, and irritable	-	Mild atrophy of cerebral cortex, ventriculomegaly with periventricular signal abnormality	*PSEN2*	c.640G>T p.V214L	E3/E3

F, female; M, Male; MRI, Magnetic resonance imaging; APOE, Apolipoprotein E genotype.

#### 3.3.1. Characteristics of an EOAD patient with APP mutation

**Figure 3:**
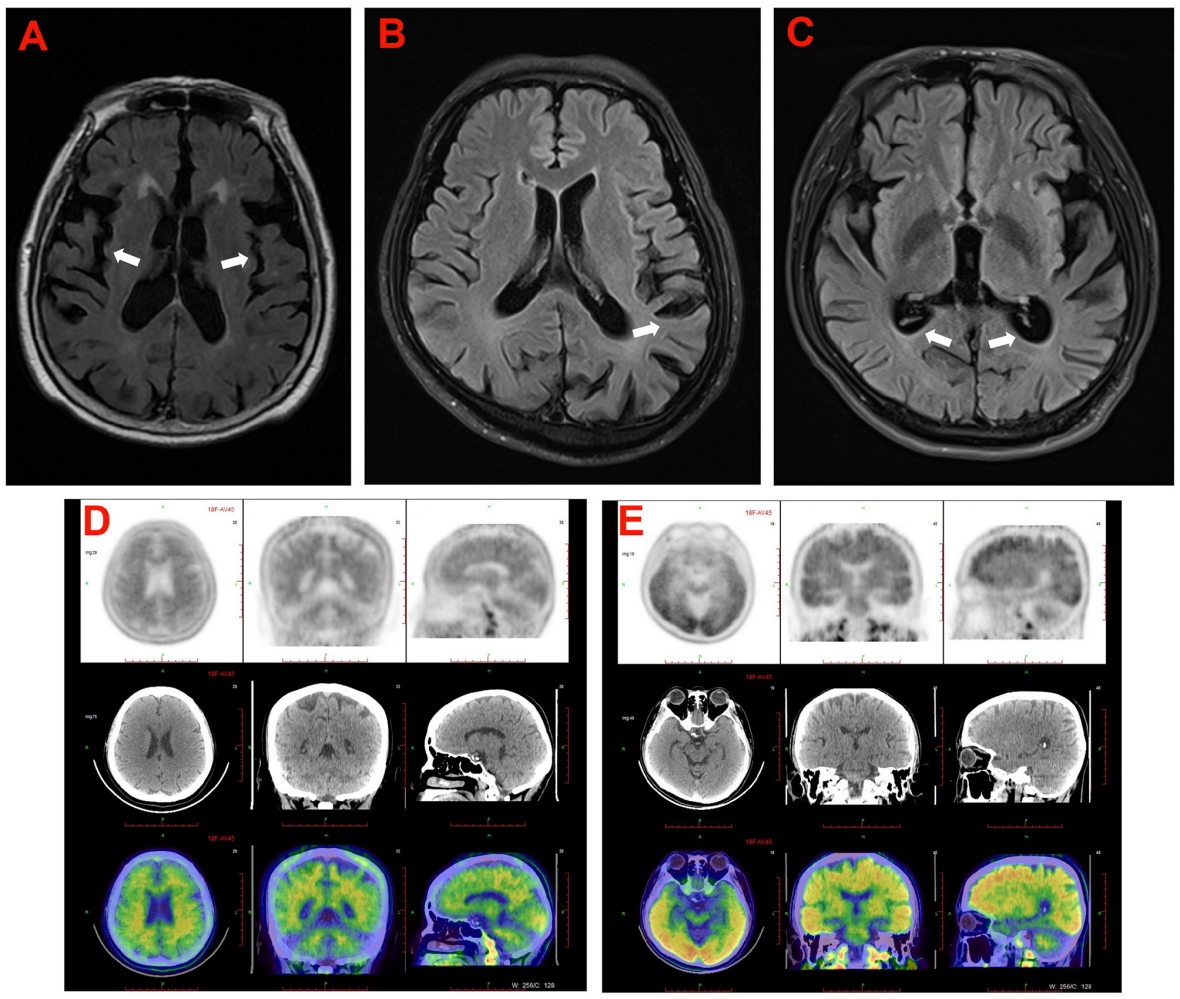
The cranial neuroimaging of the probands in the five families. **(A)** The T2 weighed image showed atrophy of cerebral cortex (arrowhead) and mild ventriculomegaly with periventricular signal abnormality in proband (II-3) in family 5. **(B)** The T2 FLAIR image showed atrophy of cerebral cortex (arrowhead) predominantly in temporoparietal lobe of the proband (II-4) in family 4. **(C)** The T2 FLAIR image showed atrophy of cerebral cortex (arrowhead) and mild ventriculomegaly in proband (III-3) of family 2. **(D, E)** 18F-AV45 brain images of the proband (II-3) in family 1 **(D)**, and the proband (II-2) in family 3 **(E)**.

Proband 1 (**II:3**) was a 53-year-old man, carrying the c.2061A > C (p.K687N) mutation in *APP*, who was initially admitted to our inpatient ward because of short-term memory impairment within 1 year. He had difficulty figuring out how to put on his clothes and finding his way to a familiar place. Involuntary resting tremors of both upper extremities appeared frequently and progressed gradually. He scored 22/30 on the mini-mental state examination (MMSE) and 20/30 on the Montreal Cognitive Assessment (MoCA). MRI scans revealed enlarged ventricles and sulci, indicating generalized cerebral atrophy. Brain AV-45 PET-CT scans showed diffuse amyloid plaque deposition in the cerebral cortex, particularly in the occipital cortical areas (Figure 3D). The levels of Aβ42 and Aβ40 in the CSF were 4.1 pg/mL (≥651) and 242.8 pg/mL, respectively, and those of total tau and phosphorylated tau were 628 pg/mL (<290) and 87.2 pg/mL (≤61), respectively. A similar onset age and memory impairment phenotype were described in his affected mother (**I:2**) and older sister (**II:2**). Genetic analysis revealed that both his mother and sister carry the same mutation.

#### 3.3.2. Presentations of EOAD patients with PSEN1 mutations

Proband 2 (**III:3**) in family 2, carrying the c.428T > C (p.I143T) mutation in *PSEN1*, was a 37-year-old man who was admitted to our department because of a 5-year history of cognitive decline. The patient initially complained of problems with recalling recent events, frequent oversights, and word-finding difficulty. After 2 months, he developed generalized seizures. His seizures were well controlled with the antiepileptic drug carbamazepine. During the following years, the cognitive disturbances progressively worsened, and he gradually became more apathetic and socially disinhibited, with less spontaneous speech. His MMSE score was 17/30 at 34 years of age. Neurological examination showed disorientation, comprehension deficits, slurred speech, and severe impairment in episodic memory and executive functions. The patient's medical record was unremarkable, except for a history of illicit drug addiction. Brain MRI showed significant temporal lobe atrophy and ventricular enlargement (Figure 3C). The family history revealed that the proband's mother (**II:4**) and older sister (**III:2**) experienced similar symptoms at 45 and 34 years of age, respectively, and his grandmother (**I:2**) had died at 60 years of age, with an onset at 48 years. Unfortunately, the proband's mother and older sister are no longer available because they have passed away. The proband's cousin (**III:1**) also had the same mutation but was clinically asymptomatic.

Proband 3 (**II:2**) in family 3, carrying a duplication mutation (c.702_704dup and p.L235dup) in the *PSEN1* gene, was a 45-year-old man who was admitted to our department because of a 2-year history of cognitive decline. The patient complained of frequent memory problems, including difficulty in word finding and remembering recent events. His daily tasks and activities appear to be affected as well. After 1 year, he exhibited behavioral symptoms such as paranoia, apathy, and irritability. The scores of the MMSE and MoCA tests were 22/30 and 18/30, respectively. Neurological examination revealed mild impairment in disorientation, spatial memory, and executive functions. Brain MRI revealed conspicuous hippocampal atrophy and ventricular enlargement. Brain AV-45 PET-CT scans showed prominent amyloid deposition in the frontal, temporal, parietal, and occipital cortical areas (Figure 3E). There was no history of AD or other neurological diseases in his family. His father and mother died from heart disease and lung cancer, respectively, before the age of 65 years. DNA testing could not be carried out because his parents' DNA was no longer available.

#### 3.3.3. Phenotypes of the patients with PSEN2 variants

Proband 4 (**II:4**) was a 54-year-old woman, carrying a known missense variant (c.505C>A, p.H169N) in *PSEN2*, who presented with fast shaking of her left leg at the age of 50 years, that had started insidiously and had progressively worsened over the last half year, eventually resulting in involuntary trembling of the whole body. The shaking occurred mainly during activities and was relieved by resting. There were no significant findings on neurologic examination at that time. Thus, an initial diagnosis of essential tremors was made. From the age of 52 years, she gradually exhibited forgetfulness, apathy, and non-fluent speech. Physical examination revealed deficits in episodic memory and slurred speech, together with pronounced tremors when holding her arms outstretched and incoordination in movements. She scored 21/30 on the MMSE test. Brain MRI revealed a prominent atrophy of the cerebral cortex, predominantly in the temporoparietal lobe (Figure 3B). Family analysis revealed that her two older sisters (**II:2** and **II:3**) carried the same variant and experienced similar tremor symptoms in their early 50s. The proband's mother (**I:2**) had similar phenotypes and died at the age of 58 years, with an onset at 45 years.

Proband 5 (**II:3**) was a 49-year-old man, carrying a heterozygous missense variant (c.640G > T, p.V214L) in *PSEN2*, who had problems with memory disturbance. In the past 2 years, he gradually became forgetful, apathetic, and irritable. Brain MRI revealed mild atrophy of the cerebral cortex and mild ventriculomegaly with periventricular signal abnormality (Figure 3A). Decreased levels of Aβ42 (339.73 pg/mL) and an elevation in tau species (p-tau 93.14 pg/mL) were observed in the CSF of the proband. There was no medical record of dementia or other neurological disorders in his family members. His father and mother refused to take genetic testing.

#### 3.3.4. Phenotypes of the patients with uncertain significance in other AD-risk genes

We identified three different variants of uncertain significance in *GRN* and *SORL1*. Of these variants, the c.453del (p.M152Cfs^*^104) variant in *GRN* was found in a sporadic AD case, who developed memory disturbance and apathy at the age of 54 years. The other *GRN* variant, R564C, was identified in a sporadic patient who was diagnosed with AD at 62 years of age. It is noteworthy that the SORL1 variant, c.4384T > G (p.F1462V), has been identified in a sporadic case with AD who became forgetful at the age of 47 years and progressed to dementia at 51 years of age.

## 4. Discussion

Early-onset Alzheimer's disease represents a highly genetically heterogeneous group of progressive dementia that overlaps in its clinical presentation with LOAD. Characterization of the clinical and genetic determinants of EOAD is crucial for fully understanding the etiology of AD and developing more effective targets for prevention and treatment. In our EOAD cohort, we found that 40% (2/5) of familial EOAD and 7.69% (1/13) of sporadic EOAD patients carried pathogenic or likely pathogenic variants, including a known pathogenic *PSEN1* mutation and two novel pathogenic mutations in *PSEN1* and *APP*, respectively. We also detected two known VUS in *PSEN2* (p.H169N and p.V214L). In addition, we found some rare variants in several genes, either causal for other types of dementia or previously identified as risk factors for AD, suggesting the possible involvement of multiple genes in the etiopathology of AD. The two variants, *GRN* R564C and *SORL1* p.T1513N, may be risk factors for AD clinical phenotypes. Based on our findings, the majority of cases (at least 13/18, 72.22%) in the cohort still remain genetically unexplained, which may also contribute to the missing genetic etiology of EOAD.

The mean AAO (44.8 ± 7.9 years) in our patients with identified mutations in *APP, PSEN1*, and *PSEN2* was similar to that previously described in a Chinese cohort (18). Our study verified that families carrying *PSEN1* mutations have an earlier mean AAO than those with *APP* or *PSEN2* variants. In the present study, we identified a novel *PSEN1* mutation, p.L235dup, associated with EOAD. The mutation frequency was absent in any public databases, indicating that this mutation is very rare. Unfortunately, both parents are not available for genetic testing in this case because they have passed away. Remarkably, three known missense mutations (L235V/L235P/L235R) at the same residue of *PSEN1* have been previously reported in patients with EOAD in different populations (19–21). Yang et al. (22) reported that transgenic mice expressing *PSEN1*-L235P showed an increased production of Aβ, together with increased phosphorylation of tau and synaptic protein loss. Therefore, based on the 2015 American College of Medical Genetics and Genomics/Association for Molecular Pathology (ACMG/AMP) guidelines, the p.L235dup mutation was classified as pathogenic. In addition to having an earlier onset, patients with *PSEN1* mutations more frequently manifested atypical cognitive symptoms and additional neurological features (behavioral change, language impairment, dyscalculia or executive impairment, myoclonus, seizures, pyramidal, extrapyramidal, or cerebellar signs) when compared to *APP* mutation carriers (12). It was reported that *PSEN1* mutations occurring after codon 200 were more frequently associated with spastic paraparesis and visuospatial impairment as well as an earlier AOO, while mutations before codon 200 were more frequently associated with seizures and myoclonus (23, 24). In our cohort, the proband with the p.I143T mutation developed seizures and bradykinesia in his 30s, while the p.L235dup mutation carrier exhibited typical patterns of memory loss and behavioral change. The known mutation in *PSEN1* p.I143T has been found in several families worldwide, with the age of onset between 30 and 41 years, but clinical phenotypes of mutations were different in these families (15, 25, 26). Most of these carriers presented with typical AD symptoms, including memory impairment, disorientation, dyspraxia, and dysphasia. In the later stages of the disease, patients with this mutation may develop movement disorders (limb apraxia, rigidity, dystonia, myoclonus, or seizures) as well as paranoid delusions, hallucinations, and aggressiveness. However, our patient with the same mutation initially presented with seizures for 3 months, followed by apraxia and rigidity, which were rarely reported in the early stage of the disease or as initial symptoms. Together, our data suggest that *PSEN1* mutations are associated with very early disease onset and a variable phenotype.

Unlike *PSEN1* carriers, mutations in *PSEN2* cause AD with milder phenotypes and later onset age (27). Some *PSEN2* mutation carriers may exhibit variable clinical phenotypes of AD. The known variant in *PSEN2*, p.H169N, has been found in several individuals of East Asian ancestry (15, 28, 29). The variant was classified as VUS, with higher frequency in a much higher population frequency in East Asian ancestry. It is essential to establish the relationship between the *PSEN2* variant, p.H169N, and AD; functional assessment of this variant may be required. The p.H169N carriers were reported to experience typical AD symptoms with memory decline at the beginning. In this study, we present an EOAD family carrying the p.H169N variant in which, on initial presentation, the affected individuals had symptoms of uncommon action tremors. Symptoms such as forgetfulness, apathy, and speech impairment developed later during the development of this disease. Although rare, extrapyramidal signs may also be observed in the early stage of EOAD or as initial symptoms. Our findings extend the current knowledge of the phenotypic heterogeneity of EOAD families carrying the *PSEN2* variant, p.H169N. In addition, the *PSEN2* p.V214L variant has also been reported in many East Asian ethnic groups, with prominent memory impairment and visuospatial deficits which commonly occur between 48 and 69 years of age (15, 17, 30, 31). Although structural changes were predicted *in silico*, the pathogenicity of the *PSEN2* variant, p.V214L, associated with EOAD was questioned, based on its much higher frequency in the East Asian population. This variant was reported as either disease-causing with reduced penetrance or as a risk factor for EOAD (32). Therefore, functional assessment of the variant (*PSEN2* p.V214L) is required to establish its pathogenicity in EOAD.

The heterozygous mutation in *APP* (c.2061A>C, p.K687N) was first identified in our study, and this mutation was found to co-segregate with the disease in family 1. Another variant (c.2061A > T, p.K687N) affecting the same amino acid sequence of *APP* was previously reported as a pathogenic variant in a German EOAD family, which exhibited a similar phenotype to the family in our study (33). *In vitro* functional studies provide some evidence that the p.K687N mutation may have a damaging effect on protein function (33). The p.K687N mutation was not classified by Alzforum and this variant was previously observed in a single-affected family without any evidence of co-segregation. Moreover, a known mutation, p.K687Q, at the same residue has been previously reported in two unrelated Chinese EOAD families (18, 34). Thus, this variant was re-classified as a pathogenic/likely pathogenic variant, based on the 2015 ACMG-AMP criteria. In addition to a decline in progressive memory, other rare motor symptoms, such as rest tremors, were also observed in our patient during the course of the disease. Scarmeas et al. (35) found that motor decline, for example, in speech/facial expression, rigidity, bradykinesia, or posture/gait occurs frequently and progresses rapidly in AD, but that tremors were less frequent throughout the course of the disease. To the best of our knowledge, isolated rest tremors have not yet been reported in patients with *APP*-related EOAD. Our findings expand the phenotypic spectrum of *APP*-related EOAD.

There were still some limitations to this study that need to be noted. First, we failed to perform screening of copy number variation (deletions or duplications) in the prion protein gene (*PRNP*), *C9ORF72* (chromosome 9 open reading frame 72), and *APP*, which might be responsible for a proportion of EOAD and other types of dementia (36–38). The second limitation is the lack of functional assessment of the potentially pathogenic variants. Third, it should be noted that intronic variants might participate in the genetic determinism of both familial EOAD and sporadic forms. For negatively screened families and sporadic cases, whole-genome sequencing may be helpful for the identification of additional causative genes and for expanding our understanding of genetic mechanisms in EOAD.

In summary, our study provides further evidence of the genetic profile of patients with EOAD from China and expands the mutation spectrum of both *PSEN1* and *APP*. In addition, our results highlight the clinical heterogeneity in patients with EOAD and mutations in *PSEN1, PSEN2*, and *APP* and suggest the strong effect of genetic variants on clinical phenotypes. Next-generation techniques provide a useful means to investigate the genetic determinants in clinical cases showing heterogeneous clinical presentation and reduced penetrance. Future functional studies are needed to clarify the interaction between causative gene mutations and phenotypic heterogeneity.

## Data availability statement

The data presented in the study has been deposited in the DDBJ repository, https://getentry.ddbj.nig.ac.jp/, accession numbers LC756953 and LC756954.

## Ethics statement

The studies involving human participants were reviewed and approved by Ethics Committee of Wuhan Union Hospital. The patients/participants provided their written informed consent to participate in this study. Written informed consent was obtained from the individual(s) for the publication of any potentially identifiable images or data included in this article.

## Author contributions

ZLia and YW: data acquisition and analysis, visualization, and writing the original draft. ZLiu and CL: study design and conceptualization, data analysis, writing–review and editing, and funding acquisition. All authors contributed to the article and approved the submitted version.
